# COVID-19 Presentation in Association with Myasthenia Gravis: A Case Report and Review of the Literature

**DOI:** 10.1155/2020/8845844

**Published:** 2020-08-17

**Authors:** Elif Aksoy, Turgut Oztutgan

**Affiliations:** Department of Pulmonology, Kolan International Hospital, Merkez, Kaptanpaşa Mahallesi Okmeydanı Kavşağı, Darülaceze Cd. No. 14, 34384 Okmeydanı Şişli, İstanbul, Turkey

## Abstract

Currently, there are scarce data on how COVID-19 affects people with myasthenia gravis. Theoretically, there is a higher risk of experiencing severe manifestations of COVID-19 due to the common use of immunosuppressive drugs and potential respiratory failure in relation to respiratory muscle weakness. This is one of the early cases of COVID-19 reported in association with myasthenia gravis. Here, we highlight the prognosis, discuss the pathophysiological mechanisms, and prompt the consideration of convalescent plasma therapy in myasthenia gravis patients with concomitant COVID-19.

## 1. Introduction

The pandemic of COVID-19 is a potentially severe acute respiratory infection caused by severe acute respiratory syndrome coronavirus 2 (SARS-CoV-2). The fatality rate is 2-3% although in hospitalized adult patients this rate can rise up to 11%. Higher morbidity and mortality rates are seen among the elderly and those with underlying comorbidities and immunologic deficiencies [1]. Immunosuppressive agents, dysregulated immune responses, and respiratory muscle weakness are some of the possible etiologies that may increase the severity of outcomes related to COVID-19. Disruption of immune and inflammatory processes is a shared mechanism in both diseases [2, 3]. Initial cases of myasthenia gravis (MG) with simultaneous COVID-19 showed variable outcomes with the potential for dangerous exacerbations [2, 4]. Little is known about the association between two diseases. Here, we hope to illustrate the COVID-19 prognosis in MG patients along with the shared features of both diseases and discuss therapeutic approaches to maximize the outcomes for this special patient population.

## 2. Case Report

A 46-year-old woman with a past medical history of acetylcholine receptor antibody-positive MG presented to the hospital with fatigue in the last 3 days. Her exacerbations generally consisted of dysphagia alone. She underwent thymectomy in 2016 and maintained on pyridostigmine 60 mg twice daily for 4 years. Due to a history of close contact with a known corona-positive patient, a nasopharyngeal specimen was obtained immediately. Initial chest CT revealed no pathological abnormalities and the patient was sent home for self-quarantine.

**Figure 1:**
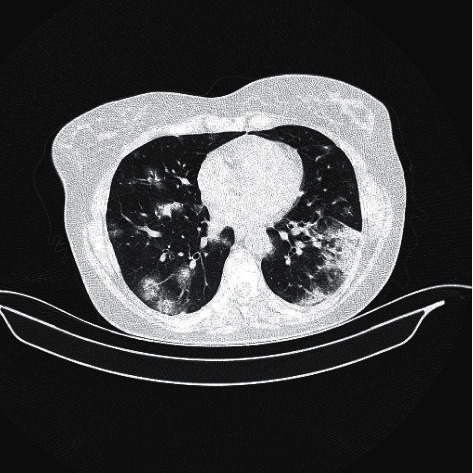
Axial CT image shows bronchocentric and nodular organizing pneumonia patterns and ground-glass opacifications.

Three days later, the patient was hospitalized after COVID-19 was confirmed by real-time polymerase chain reaction (RT-PCR). She had no other complaints but fatigue. New chest CT showed bronchocentric and nodular organizing pneumonia patterns and ground-glass opacities consistent with COVID-19 pneumonia (Figure 1). Physical exam revealed a blood pressure of 126/64 mmHg, a pulse of 104 bpm, a temperature of 38.8°C, an oxygen saturation (SpO2) of 95%, and a respiratory rate of 18/min. Laboratory results were notable for increased C-reactive protein and white blood cells up to 62 mg/L and 14 *µ*L, respectively, as well as lymphopenia of 13%. On chest auscultation, rales were detected in bilateral lower lobes on inspiration. Neurological examination was unremarkable. Pyridostigmine, favipiravir, meropenem, oseltamivir, hydroxychloroquine (400 mg twice daily first day, afterwards 200 mg twice daily), and subcutaneous low-molecular-weight heparin were started upon admission. Pyridostigmine dosage was gradually increased from 60 mg twice daily to four times daily in the consecutive days.

On day 3, oxygen therapy via nasal cannula (2-3 L) was added after the patient developed dyspnea.

On day 5, hydroxychloroquine was ceased after neurology consultation, but linezolid and intravenous methylprednisolone (40 mg daily) were added to the regimen.

On day 6, the patient had 84% oxygen saturation on oxygen therapy via mask (10 L), so she was transferred to the intensive care unit for intubation. In the meantime, a plasma donor was found. The patient received convalescent plasma therapy instead and did not undergo intubation following the immediate improvement after plasma therapy infusion. On day 10, the patient was discharged back to the inpatient floor as the symptoms resolved, biochemistry results improved, and oxygen demand was decreased.

**Figure 2:**
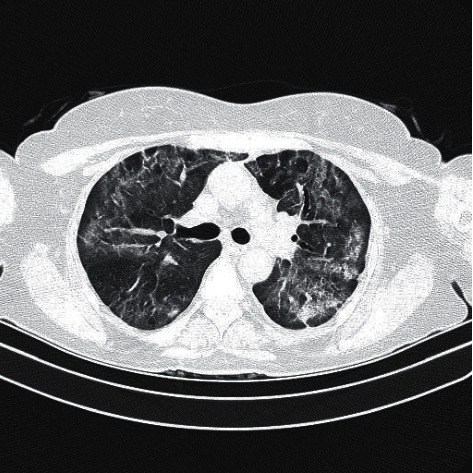
Control CT image shows partial absorption of the organizing consolidations and architectural distortion with the formation of fibrous bands.

On day 22, SARS-CoV-2 was negative on RT-PCR, but on the contrary, chest CT showed aggravated infection with partial absorption of the organizing consolidations and architectural distortion with the formation of fibrous bands (Figure 2). Because the patient had a complete clinical recovery, she was ultimately discharged from the hospital. She is called for follow-up one month later.

## 3. Discussion

COVID-19 demonstrates a relation with the nervous system, but concerning literature is scant. Thus far, headache, seizures, stroke, myalgia, and Guillain–Barré syndrome are some of the complications that have been reported in relation to COVID-19. Patients with neuromuscular diseases are considered to be more prone to developing severe forms of COVID-19. Discussions on MG patients experiencing severe complications of COVID-19 center upon risk related to bulbar and respiratory weakness along with immunosuppressive therapies although the associating mechanism is yet to be established [5]. Prolonged intensive care may also worsen the functional prognosis of these patients; therefore, a preventive approach is essential for MG patients [6].

SARS-CoV-2 presents with increased proinflammatory cytokines and chemokines along with B- and T-cell depletion. In particular, the depletion of CD8+ T cells and increased levels of TNF-*α*, IL-6, and IL-10 are correlated with increased severity of COVID-19 [7]. It is also suggested that a possible rebound trigger could be induced by autoimmune response besides the detrimental effects of the virus in the organs and immune system stimulation when there is a delay between the complications and the initial symptoms. The theory is based on the development of allo- or autoantibodies to ACE-2 receptors, the viral entry point, as it is in SARS-COV but with a higher affinity for ACE-2 in SARS-CoV2 [8].

Infections such as viral upper respiratory infection are the most common cause of respiratory failure related to myasthenic crisis [9]. Loss of self-tolerance is the pathogenesis underlying MG which is described as dysregulation of T regulatory cells. Thymectomy may be needed in some patients as in our case. Having different properties from the T lymphocytes of young healthy individuals is associated with postthymectomy status [10]. MG patients also have elevated levels of proinflammatory cytokines such as IL-6, IL-17, and IFN-*γ*. Common elevation of IL-6 is seen in both COVID-19 and MG and it is particularly associated with increased mortality in COVID-19 [11].

MG attacks can be exacerbated by drugs such as chloroquine, hydroxychloroquine, and azithromycin. Chloroquine and hydroxychloroquine are commonly used in patients with COVID-19 and as prophylaxis without any proven benefit. Both drugs are known to result in potential neuromuscular side effects [5, 6, 12]. The combined use of hydroxychloroquine and azithromycin in an MG patient with simultaneous COVID-19 is believed to cause myasthenic crisis [2]. Similarly, hydroxychloroquine may be the reason behind our patient's deterioration. Currently, there is an ongoing randomized trial that aims to clarify whether a combination therapy consisting of hydroxychloroquine and azithromycin can shorten the duration of hospitalization via anti-inflammation/immune modulation, antiviral efficacy, and preemptive treatment of suprainfections. However, patients with MG were excluded from this trial due to potential side effects [13]. Unless these drugs become the standard treatment in COVID-19 management in the future, their use should likely be avoided or be limited and require great caution in myasthenic patients [4].

In a meta-analysis, corticosteroid use in subjects with SARS-CoV-2 infection was related to delayed virus clearing and did not significantly improve survival [14]. Additionally, tapering of immune-modulating medications is shown to trigger MG exacerbations while abrupt interruption of corticosteroids may result in adrenal insufficiency [6, 9]. However, corticosteroids may be beneficial in subduing the immune response in the setting of ARDS and thus can dampen excessive lung damage. They should be used with caution in MG patients.

Monoclonal antibodies such as eculizumab and tocilizumab are used in the treatment of refractory MG and are promising candidates in the management of cytokine storm leading to ARDS related to COVID-19 [6, 15]. They were used experimentally in COVID-19 and shown to be successful in treating patients who had either COVID-19-related severe pneumonia or ARDS [4, 15].

Our patient greatly benefited from convalescent plasma therapy. Despite the suspicion surrounding the convalescent plasma use, an increase in survival rates was observed during the pandemics of SARS and MERS when conventional treatment failed. Also, a systemic review and a meta-analysis to evaluate the clinical effects of convalescent plasma showed a statistically significant reduction in mortality in COVID-19 patients [16]. Besides the neutralizing antibodies, other proteins such as anti-inflammatory cytokines, clotting factors, natural antibodies, defensins, and pentraxins also exist in the serum of the donors. IVIG is the first-line therapy in some autoimmune diseases such as Guillain–Barré and effective rescue therapy in MG along with plasmapheresis in severe cases or acute exacerbations of MG [17]. Improvement was shown after IVIG infusion in new reports of MG patients. This is especially important for MG patients who can be considered as high risk given the potential for respiratory muscle weakness. Patients who had MG exacerbations characterized with dysphagia prior to infection or developed dysphagia after being infected with the virus had complete resolution of symptoms after being treated with IVIG [2, 4]. However, one patient had complete recovery only after undergoing plasmapheresis following IVIG as her swallowing functions did not improve with the latter [18]. Given these terms, transfusion of IVIG may take part in immunomodulation by amelioration of severe inflammatory response and hence ease down the hyperinflammation or “cytokine storm” driven by proinflammatory cytokines [17, 19]. Targeting complement-mediated immune response was shown to be beneficial in both diseases. Plasmapheresis can reinforce the therapeutic effects when IVIG is insufficient for complete recovery.

Patients with muscular dystrophies who have ventilatory muscle weakness are more prone to experience severe complications of COVID-19 and may not be able to return to their baseline after the infection [5]. Similarly, our patient had worsened radiologic images contrary to complete recovery on discharge from the hospital. Overproduction of proinflammatory factors along with reactive oxygen species can cause ARDS and result in pulmonary fibrosis [20]. Amplified immune response pertaining to a mutual reinforcement between MG and COVID-19 may have caused permanent lung damage in our patient.

## 4. Conclusion

Amelioration of severe inflammatory response is a shared feature of the therapeutic process in MG and COVID-19. Thus, immunomodulatory therapies such as convalescent plasma therapy in our case were particularly beneficial in the management of ARDS following COVID-19 with simultaneous MG. However, we suggest individualized therapy considering the severity of neurological manifestations of MG. Also, these patients should be closely monitored during the disease course. Early treatment with immunomodulatory therapies may be more effective in terms of preventing permanent lung damage than standard first-line treatments.

## Data Availability

No data were used to support the findings of this study.
